# Gender detransition: a case study

**DOI:** 10.1111/1468-5922.12711

**Published:** 2021-11-10

**Authors:** Lisa Marchiano

**Affiliations:** ^1^ Philadelphia USA

**Keywords:** adolescence, affirmative care, detransition, gender, gender dysphoria (GD), transgender, transgenre, détransition, soins trans‐affirmatifs, adolescence, dysphorie de genre, genre

## Abstract

Within the last decade, there has been a sharp global rise in the number of young people identifying as transgender. More recently, there appears to be an increase in the numbers of young people detransitioning or returning to identifying with their natal sex after pursuing medical transition. A case is presented of a young woman who pursued a gender transition and returned to identifying as female after almost two years on testosterone. The author considers and critiques the affirmative model of care for gender dysphoric youth in light of this case.

## Abstract (fr)

Dans les dix dernières années, on a vu un accroissement considérable du nombre de jeunes personnes qui s’identifient transgenre. Plus récemment, il semble qu’il y ait un accroissement du nombre de jeunes personnes qui reviennent en arrière par une détransition, ou qui reviennent à s’identifier à leur sexe de naissance après avoir souhaité une transition médicale. L’article présente le cas d’une jeune femme qui est entrée dans le processus de transition de genre et qui est ensuite revenue à une identification en tant femme, après presque deux années sous testostérone. A la lumière de ce cas, l’auteur examine et critique le modèle de soin qui va dans le sens d’affirmer la dysphorie de genre chez de jeunes personnes.

## Abstract (de)

Innerhalb des letzten Jahrzehnts hat die Zahl der jungen Menschen, die sich als transgender identifizieren, weltweit stark zugenommen. In jüngerer Zeit scheint es einen Anstieg der Zahl junger Menschen zu geben, die sich nach einer medizinischen Umwandlung wieder mit ihrem Geburtsgeschlecht identifizieren. Es wird ein Fall einer jungen Frau vorgestellt, die eine Geschlechtsumwandlung anstrebte und sich nach fast zwei Jahren mit Testosterongaben wieder als weiblich identifizierte. Der Autor betrachtet und kritisiert das affirmative Modell der Betreuung geschlechtsbezüglich dysphorischer Jugendlicher im Lichte dieses Falls.

## Abstract (it)

Durante l'ultima decade c’è stato un netto incremento del numero dei giovani che si sono identificati come transgender. Più recentemente, sembra ci sia un aumento nel numero dei giovani che de‐transitano o ritornano ad identificarsi con il loro sesso originario dopo aver fatto una transizione medica. Viene presentato il caso di una giovane donna che si è sottoposta ad una transizione ed è poi tornata ad identificarsi come femmina dopo circa due anni di testosterone. L’Autrice considera e critica il modello di cura affermativo in relazione alla disforia di genere nel caso clinico di una giovane.

## Abstract (ru)

За последнее десятилетие мы стали свидетелями резкого увеличения числа молодых людей, которые идентифицируют себя как трансгендеров. Однако недавно наметилась обратная тенденция: де‐переход или возвращение к идентификации cо cвоим биологическим полом после медицинского вмешательства. Представлен случай молодой женщины, которая предприняла попытку смены пола, а затем вернулась к идентификации себя как женщины после двух лет приема тестостерона. В свете этого случая автор критически рассматривает аффирмативную модель помощи гендерно‐дисфоричным молодым людям

## Abstract (es)

En la última década, ha habido un crecimiento global agudo de jóvenes que se han identificado como transgénero. Más recientemente, pareciera haber un incremento en el número de jóvenes de‐transicionando o retornando a la identificación con su género natal luego de haber realizado una transición médica. Se presenta el caso de una joven mujer quien prosiguió una transición de género y retornó a identificarse como de género femenino, casi dos años después de haber tomado testosterona. La autora considera y critica, a la luz de este caso, el modelo afirmativo de cuidado para la disforia de género en las y los jóvenes.

## Abstract (zh)

去跨性别化:个案研究

在过去十年间, 全球跨性别青年的数量剧增。近期, 去除跨性别或是在追逐医学跨性别的个体重返对自身生理性别的认同的年轻人数量也在增加。文章呈现了一个个案, 关于一位女性曾经追求性别转换, 但在使用睾丸酮差不多两年后, 又重返女性化性别的认同。作者透过这个案例论述了对性别焦虑的年轻人的性别平权护理模型。

## Introduction

In the past decade, there have been significant changes in the demographics of those presenting with gender dysphoria. The number of adolescents seeking treatment for gender dysphoria has been rising since 2004, with sharp increases noted especially over the last decade. In the words of one researcher, ‘the increase in the number of adolescents referred to specialized gender identity clinics/programs has become an international phenomenon, observed all across North America, Europe, Scandinavia, and elsewhere’ (Zucker 2019, p. 1). In the last 10 years gender clinics in the U.K. have seen a 4,400% increase in teen girls seeking treatment for gender dysphoria (Rayner 2018). Also notable is that the sex ratio of those seeking services has flipped. Prior to about 2006, most adolescents presenting with gender dysphoria were natal males (Aitken et al. 2015). In subsequent years, natal females have made up the majority of referrals, with the ratio of natal female to natal male as high as 7.58 to 1, according to one calculation (Zucker 2019). Over the past 10 to 15 years, treatment for these adolescents has also undergone substantial change, for example, the use of gonadotropin releasing hormone agonists (GnRH agonists) to suppress puberty (de Vries et al. 2014) followed by cross sex hormones and later surgery: double mastectomies are being performed on trans boys as young as 13 years‐of‐age (Olson‐Kennedy et al. 2018).

Recent clinical guidelines from the World Professional Association for Transgender Health (WPATH) and the Pediatric Endocrine Society have relaxed their eligibility criteria for treatments (Coleman et al. 2012; Hembree et al. 2017). In addition, the informed consent model of care has become widespread (Ashley 2019). For example, in the United States those over 18 years‐of‐age can access cross sex hormones at one of Planned Parenthood’s informed consent clinics without input from a mental health clinician. Finally, the gender affirmative model of care has become increasingly accepted as the dominant model of care in the treatment of children and adolescents (Ashley 2020). Together with the rise of teens seeking to change gender, the number of young people detransitioning (reaffirming their natal sex) also appears to be increasing. Detransitioners are now sharing their stories online and entering therapy. Though there is still little research on this population, discussions of individual cases are available (Withers 2015, 2020; Levine 2017; D’Angelo 2020; Korpaisarn & Modzelewski 2019; Turban & Keuroghlian 2018; Marchiano 2017). Detransition has also begun receiving increased attention in the clinical literature (Butler & Hutchinson 2020, Expósito‐Campos 2021, Entwistle 2021, Guerra et al. 2020).

I feel that the rising rates of transition and detransition call for a depth understanding; this cultural phenomenon has life‐long physical and psychological effects on teens and young adults. The case of a young woman in my practice who detransitioned illustrates the complex psychological dynamics underlying the quest for gender change. This young person began to identify as trans in her teens, took testosterone for almost two years in order to acquire a masculine appearance and later reaffirmed her identity as female. Although a single case cannot represent the full range of issues involved in gender detransition, her story depicts the implications of unaddressed psychological complexities in gender transition.

## Case history: Maya

A young woman whom I will call Maya was referred to me by someone aware of my experience with gender dysphoria in young people. Maya was quickly open to a connection and we began once‐weekly treatment. A petite young woman, Maya presented in a typical, if casual manner, and usually wore jeans and a T‐shirt to our sessions. Maya’s parents were educated professionals. According to Maya, it was a standing joke in her home that her mother was not cut out for motherhood. She precipitously ended her maternity leave just two weeks after Maya’s birth to return to her demanding job, sending for an aunt to come and take care of the baby.

Maya’s aunt lived close by and Maya remembers her as warm and loving. Maya loved her traditional cooking and often slept with her because she was afraid to sleep alone. Both parents were absorbed in their careers and Maya’s memories of them are few except for the stilted family meals her mother scheduled a couple of times during the week. Mother did take an interest in Maya’s clothing and dance lessons, but it was her aunt who provided daily care. Maya remembers her father as sometimes warm and playful and other times angrily explosive. He and Maya’s aunt did not get along, and she voiced her criticism of him to Maya.

When Maya was nine, her aunt suddenly died. Maya was not allowed to attend the funeral as her parents felt that it was better if Maya didn’t ‘dwell on the loss’ and they encouraged her to ‘move on’. A succession of au pairs and babysitters quickly replaced Maya’s aunt and life went on ‘as usual’. Maya managed to maintain a strong connection to her dance squad peers until age 12 but had difficulty with the transition to middle school and the pubertal changes which caused significant breast development. She dropped out of dance and began dieting. Mother, who was slender and small‐boned was ‘concerned’ about Maya’s rounding body and encouraged dieting. Maya discovered she could binge and purge. She also began to struggle in school and was diagnosed with ADHD. She was assigned to some remedial classes and acquired a reputation as a ‘problem student’ despite her obvious intelligence. Deficits in parental attention and the loss of her aunt were augmented by these physical and school changes which together with the absence of emotional support at home left her feeling outcast and unworthy. Maya isolated and spent more and more time online.

Maya’s time on social media fed a rumination about identity. When she shared with some of her online friends that she wondered whether she might be trans because she felt uncomfortable in her body, her friends on social media were quick to affirm and celebrate her. Maya cut her hair boyishly short and began wearing oversized jeans and sweatshirts. In her online life – which felt more real and rewarding than her ‘real’ life – Maya adopted a male name. At 14 years‐of‐age, Maya announced to her mother that she was trans and wanted to see a gender therapist and begin hormone therapy. Although mother was at first dismissive, she eventually accompanied her daughter to the school psychologist. Even Maya was surprised by how quickly the psychologist confirmed her trans identity, agreed with the name change and encouraged her parents to affirm Maya’s identity as male and support medical transition. Her parents, however, would not approve this treatment. The psychologist concurred with Maya that mother’s reluctance to approve hormone treatment was harmful. However, at age 18, when Maya no longer needed parental permission, she visited an informed consent clinic and was given a prescription for testosterone after a 30‐minute meeting with a physician’s assistant.

Maya’s parents were preoccupied with work and Maya was left to navigate this tumultuous time mostly on her own. Maternal attention often came as criticism of Maya’s refusal to conform to expectations about appearance and traditional femininity as mother placed a high value on cultural norms for feminine beauty: her figure, make‐up, fashionable clothes and heels. Mother had taken pride in the elaborate outfits required for Maya’s childhood dance performances; ‘she liked to dress me up’, Maya recalled. Mother found Maya’s masculine presentation enraging, no longer being the adorable daughter who could serve as a narcissistic extension. Maya’s father attempted to be more tolerant of Maya’s gender struggles, but his efforts were sporadic and undermined by his occasional rages. Maya recalls that their relationship during her teen years was characterized by alternating currents of distance and conflict. This pattern intensified during her period of trans identification.

When Maya began taking testosterone it initially made her euphoric, however overall she found the 20 months of treatment difficult and disorienting. She became easily enraged and isolated herself in her room to shield herself from experiences that would provoke and overwhelm her. She lost contact with friends, rarely attended class and, preoccupied with matters other than academics, her grades were poor. Though she presented and often passed as male, she was small and slight and so people usually treated her as a much younger boy. When Maya was assigned a single room in an all‐male dormitory, she felt very vulnerable among the bigger men and showered late at night to avoid them. She dropped out of college at the end of her first year.

Maya’s mental health had deteriorated, possibly due in part to testosterone. Although there is little research about the effects of testosterone in natal females, Maya feels that the hormone destabilized her emotionally. She recalls being an ‘anxious, angry mess’ and experienced intense self‐destructive moods. She was hospitalized twice, once for suicidal intent and once because depression rendered her unable to bathe or care for herself. A few months after her last hospitalization, Maya stopped taking testosterone because she suspected that the hormone was contributing to her worsening mental health. Shortly after that, she began the process of re‐identifying as female, and six months later, Maya entered therapy with me due to continued struggles with depression, anxiety and disordered eating.

## Discussion

At first, I didn’t know what to make of Maya. She swung back and forth between self‐states and narratives. Some weeks, she was full of passionate energy about a new project, feeling expansive and optimistic. Other weeks, she was overwhelmed with emotions and spoke in a pressured and hyperbolic manner. For the first several months of treatment, she brought a new, overwhelming problem to every other session. One week, she was insistent that her ADHD would forever prevent her from attending community college, another week, she would be paralyzed by indecision over whether or not to move to another city. She continued to struggle with bulimia. Sometimes she expressed confidence that things were getting better, other times she confessed to bingeing and purging nearly every day. Despite occasional bright moods, emptiness and sadness underlay most of her experiences. Maya didn’t know who she was and neither did I.

In trying to formulate an understanding of Maya and her suffering, I would catch hold of a narrative thread, only to have it ripped out in a subsequent session. We couldn’t settle on anything cohesive that might contain, much less organize, Maya’s chaotic emotional life and missing sense of identity. Maya’s inability to symbolize her experience provoked a concretizing countertransference. I found myself wanting to contain her pain by locating it within a single explanatory narrative that we could then neatly ‘conquer’ with appropriate treatment. Was the main issue the eating disorder? Then it might be addressed by a referral to a specialized treatment centre. Or was her ADHD the main cause of her confusion and disorientation? Then maybe she could be helped by a coach who could teach her appropriate skills. The impulse to contain by diagnosing and treating Maya’s distress arose from a sense of disorientation and inadequacy that was alive in the field between us. Maya sometimes felt as though she were ‘too much’ for me and I worried that I wasn’t enough for her. This led me to look for a solution ‘out there’, beyond the bounds of our relationship. I frequently felt overwhelmed and pulled to offer concrete suggestions in a desperate attempt to relieve distress and create a sense of order. I wondered if this echoed the dynamic between Maya and the school psychologist who had so quickly affirmed her trans identity. In both cases, ‘the emphasis … was on doing something, fixing targeted symptoms’, in the words of Laurel Silber (2019, p. 138).

## Unmetabolized grief

After about six months, Maya off‐handedly referenced the loss of her aunt. I was surprised to learn of this significant rupture, and in such a casual manner, and reflected this to her. This began a period of our work in which we explored this important early relationship. In the analytic container, Maya was able to recall many details of the relationship that she hadn’t remembered for years. Importantly, Maya had few memories of her aunt’s death, and almost no access to any feelings around this event until we began to explore it together. She was surprised by the strength of her emotion as she recalled her aunt’s loving presence. When she died, the secure base of love and care that she had provided was gone overnight. Maya was alone with only babysitters to ‘mind’ her. Without any way to integrate this significant trauma into consciousness, the pain, grief, fear, and confusion Maya felt went into her body, for it had nowhere else to go. Relational psychotherapist Laurel Silber speaks of ‘disarticulated grief’ and explores the ways in which such grief may manifest as gender dysphoria. In examining attachment ruptures, she writes:

The co‐creation of nonmentalizable states of mind, or the shared dissociation, serves to reduce the threat of loss of connection to attachment figure(s), lonely as that may be. To protect from thinking, disavowed affects can move within the concrete realm of the body and find expression in unsymbolized felt experience …. Disavowed affects find hosts: one of them, located in gender.

(Silber 2019, p. 136)

Maya’s adolescent gender dysphoria may therefore have been, at least in part, her psyche’s way of giving expression to split‐off and unformulated grief related to significant attachment ruptures, especially the loss of her aunt and possibly also the significant rupture that occurred at two weeks of age when her mother abruptly went back to work This latter loss was not something that we focused on explicitly, perhaps because it was so deeply unconscious that we both ‘forgot’ it. The loss of her aunt was more available to us in our work, but the earlier loss of her mother percolated beneath this, remaining unthinkable.

Australian psychoanalyst Roberto D’Angelo (2020) has written about a case of a young woman with significant trauma who transitioned in adolescence. During the analysis, she realized that her transition had been an attempt to dissociate from painful affects. ‘While medical/surgical transition seemed life‐saving at the time, it also drained Josh’s pain of any meaning, history and signification, simply encasing it in the body’ (p. 17). In such a case, transition may reinforce the reductive notion that psychic pain is located in the body and can be gotten rid of by ‘fixing’ the body. As one detransitioned woman who had a mastectomy at 23 years‐of‐age noted, ‘it felt like I had asked a doctor to just cut off one of my fingers because I thought that finger was where the depression was stored’ (personal correspondence).

## The body

Jung noted the way in which the body can become a receptacle for split‐off and denied contents. He noted that ‘the body is often the personification of the shadow of the ego. Sometimes it forms a skeleton in a cupboard and everybody wants to get rid of it’ (1950, para. 40). Jung’s prescient awareness of the modern desire to escape or alter the body as a way of avoiding contact with reviled, instinctual parts of ourselves may be relevant in cases where there is somatized distress such as eating disorders, cutting or gender dysphoria.

Maya’s eating disorder and subsequent trans identification seem likely to have been part of an effort to relegate intolerable affect to the body. Pursuing transformation through disordered eating and then gender transition had the effect of concretizing her emotional losses. Displacing painful losses onto her body seemed to allow Maya to avoid her intolerable grief and gain the illusion of control. Transition into the masculine may have been an attempt to compensate for an unbearably vulnerable aspect of her wounded feminine self. Clinicians at the Tavistock Gender Identity Development Service in the U.K. have noted this defensive dynamic among young people who desisted from a trans identity without pursuing medical intervention. One such young person felt that ‘her (female) self had carried these painful experiences which needed to be got rid of; she reflected, “I felt that I had always wanted to put that poor girl in a box and put the lid on top”’ (Clarke & Spiliadis 2019, p. 347).

## The mother complex

Throughout our work, Maya’s narrative about her mother shifted dramatically from week to week. Sometimes Maya extolled her mother’s extensive professional successes or expressed gratitude for material support, equating it with maternal care. She would describe mother’s strength and resilience as if it were a heritable quality that she could someday claim for herself. At other times, Maya was filled with explosive rage at her mother. She avoided visiting her mother for months at a time and sometimes blocked her phone calls and texts. During these phases Maya seemed to inflate her mother’s negative qualities, as if only intensified experience could have any hope of impact. But when the COVID‐19 lockdown disrupted aspects of Maya’s life, she turned to her parents, especially her mother, for emotional and material support. She began visiting her parents weekly and took a job as a receptionist at her mother’s firm. This increased contact drew her closer to her family and painful truths became unavoidable.

Maya became more distraught than I had yet seen her. Without at first knowing why, she became mired in self‐loathing. Suicidal feelings frequently overtook her and bulimia aggressively reasserted itself. I shared my impression with her that her worsening distress was a result of confronting the reality of her relationship with her mother. Daily contact with her mother for the first time in years meant that Maya had to come to terms with her mother’s relational unavailability. Maya also began to explore her relationship with her deceased aunt, seeking photographs and speaking to extended family members about the person who had been so important to her. She also reconnected with an older family friend who was able to confirm and add to Maya’s impressions about the quality of her early relationship with her mother. She validated Maya’s experience of her mother’s critical, cool and disinterested nature, and recalled that after Maya’s aunt’s death, Maya was sometimes sent to her house for the weekend. Maya would cry when it was time to leave and would beg this kindly friend not to make her go. ‘Your house feels like home and mine doesn’t’, she would say. However, this friend was able to hold the dual truths of Maya’s mother’s complex nature, the lack of maternal warmth *and* her positive qualities of intelligence, energy, will and achievement. Maya, in turn, slowly began to integrate split images of her mother – and herself.

Maya’s early attachment experiences included the secure base that her aunt had provided. I believe this allowed her to establish trust in our relationship. Alongside the experiences of confusion and disorientation in which we sometimes found ourselves, I was aware very early on of a positive transference and countertransference. I liked Maya very much and recognized that I held the positive mother for her in our work. I found Maya charming, intelligent and funny. She could make me break out into a hearty laugh – and I noticed her watching me laugh, a tentative but delighted smile on her face. I imagined it must have felt wonderful to see me mirroring and enjoying her. Over time, our connection solidified. I became less anxious in the face of her distress and she became able to use our sessions to discharge overwhelming affects in a way that made her feel more settled and contained. During one discussion about managing a difficult and expensive situation without parental help, I commented that this was the kind of thing with which many parents would help a young adult child. Maya looked at me and asked, ‘Could you adopt me?’ I heard the poignant question behind her humorous query. Maya had been looking for a good mother her whole life – first in her aunt, then in the family friend, and now in me. Though the question elicited a twinge of anxiety on my part due to the implied transgression of therapeutic boundaries and the possibility of excessive dependence, the ‘good mother’ was generally a congruent place for me in our work. I felt very warm toward Maya and enjoyed my role as her ‘adoptive’ mother.

## Confronting regressive tendencies

Maya began to see that internal factors repeatedly lured her into hoping that ‘this time’ things would be different with her mother. She had ‘relentless hope’ that she would find the mother for whom she had been yearning. This dynamic came to light in the following dream:

I’m at my parent’s house and it’s very late at night, so I decide to sleep there. I am so tired I can barely drag myself upstairs to my room. It feels almost as if I have been drugged. I have to push past a mountain of boxes piled outside my room. As soon as my head hits the pillow, I’m out, but only for a second, because my mom is there and she is shaking me awake. I’m begging her to let me sleep. She’s really strange and creepy. Her voice is excited but breathy and threatening. Her eyes are wide and glazed over. Her body language is weird and exaggerated. I don’t remember what she is saying but it is demeaning and aggressive. I start pleading with her to stop. She’s almost acting as if she is possessed. I’m telling her how much I need her love, how I’ve always admired her and wanted to be like her. Then I realize something – I think she is drunk. ‘Are you drunk?’ I ask. Her demeanour changes immediately. Instead of seeming drunk or high, she becomes intense, predatory and sinister. She looks at me and I am terrified. I regret asking her if she was drunk. She grabs my arm and I try to pull away but her grip is so strong, I feel as though she is going to break my bones. I’m begging her to stop. She brings my arm to her mouth and takes a huge, grizzly, excruciating bite out of my flesh.

We agreed that the dream showed Maya’s tendency to fantasize about her relationship with her mother by crawling back into a childlike, unconscious state. The dream illustrated that surrendering to this regressive attitude led to meeting her mother complex in its worst and most devouring form. Paradoxically, the negative mother complex in this dream also wants her to wake up, perhaps to confront the reality of the relationship.

## Envy and competitiveness

As we spent more time discussing Maya’s relationship with her mother, it felt like we had broken through a somewhat histrionic defensive layer and dropped into more authentic affect. Maya spoke more about her mother’s critiques of her adolescent body and the diets she prescribed. She talked about her mother’s disregard for her academic struggles and disdain for her subsequent lack of career achievement. One week, Maya expressed feelings of distress over her inability to decorate her new apartment. Although her mother had recently stepped in to ‘help’, Maya felt shamed. This led Maya to realize that her mother’s unspoken message was that while she excelled at many things, especially successful womanhood, Maya did not. Maya felt she had flunked weight, appearance, academics, and now aesthetics; she would never match her mother. Maya began to discover that her desire to transition was in part an angry and rebellious response to her mother. She had sought instead to identify with her father, but he also was treated with disdain, so Maya felt as if she was in a no‐win situation. As we explored themes of envy and competition it became clear that Maya was never to surpass or challenge her mother; we understood that her poor school performance and male presentation actually ‘meant’ that Maya was following the unconscious rules of the family.

## Split‐off aggression

During our discussions of envy and the taboo against surpassing her mother, I referenced ‘Snow White’ and, to my surprise, Maya was unfamiliar with the story. Snow White’s envious queen mother cannot tolerate a challenge to her beauty and therefore wishes to kill the daughter blooming into womanhood. Snow White’s innocence complex is so strong that she is unable to see through the queen’s repeated efforts to trick and kill her. Because she is not able to access her aggression, she is easily seduced by the queen into compliance. Maya’s dream of her mother biting into her flesh perhaps shows the extent to which this aggression was split‐off and held by the negative mother, Maya being unable at that point to consciously own it. Maya, like Snow White, must now take a bite of the apple and metabolize some of her mother’s poison before she can wake up and differentiate from her mother.

Maya was captivated by the tale of the vulnerable maiden who was able to grow into her aggressive capacity. Maya’s nightmare and the fairy‐tale image of metabolizing poison took hold quickly: she discovered that she could anonymously troll her mother on social media and fire her up over pet political topics. It gave Maya devilish pleasure to see her mother lose her cool. On dark days when she was feeling suicidal this activity fueled Maya with a sense of agency and forward momentum. I felt this use of trickster aggression gave Maya a toe‐hold of power in the relationship with her mother. Overall, I felt that this was a positive development that could mature into claiming her authority overtly in time.

## Sacrificing emotional reality

According to Ronald Fairbairn (1943), the traumatized child introjects an idealized parent and a persecutory or absent parent and relates to them out of two corresponding self‐states. For Maya, relating to the idealized mother offered the illusory promise of love and care, but required her to sacrifice relational truth. Maya recalls trying to tell other adults about her mother’s indifference and coldness and always being reassured that her mother loved her. This dynamic now became visible in her adult relationship with her mother. When she looked forward to a weekend get‐together with mother, Maya began to see that she was silencing an awareness of reality: the meeting would be hurtful or disappointing, and it would end with a confirmation of her mother’s inaccessibility. This is the bargain she had made repeatedly in the past – that her own emotional truth would be sacrificed in exchange for the hope of maternal love and attention. We came to see a similar dynamic in her identification of herself as ‘trans’: Maya had sacrificed her female ‘identity’ in exchange for belonging and connection in the trans community.

As the dynamics with her mother came into focus, we had a clearer understanding of the defensive function of Maya’s trans identity which seemed to serve several unconscious goals. Rejecting her femaleness offered Maya a way of rejecting her mother and asserting her independence and defiance of her mother’s expectations. This allowed Maya to disavow her desire to be like her mother (which included identifying as ‘female’) and be loved and admired by her while also allowing her to be the obedient child by abiding by the unwritten rule that she must never surpass her mother. It was a way to opt out of her mother’s paradigm of feminine attractiveness, and it gave Maya a way to identify with the aggressor by acting out her rage towards her own body. Just as her mother imposed restrictive dieting regimes on Maya’s growing body to change it and make it conform to her arbitrary expectations, Maya imposed testosterone and binding her breasts to change her body and make it conform to *her* arbitrary expectations. Identifying as trans also shifted the power dynamic in Maya’s relationship with her mother, allowing her to claim some aggression and authority. When Maya tried to speak about her difficulties with her mother as a child, her concerns were often dismissed or minimized. Now, as the righteous, beleaguered victim of her mother’s intolerance and lack of care, adults and peers agreed loudly that Maya’s mother was deficient. As a trans man, Maya’s complaints about her mother were valorized.

## Gender affirmative approach

When Maya sought therapy from the school psychologist, it seems that she was treated according to the gender affirmative model of care. The therapist affirmed Maya in her belief and wish to identify as trans and confirmed her belief that she needed to transition socially and medically. The gender affirmative model of care was first developed in the U.S. and has become the dominant way of working with trans identified teens in many contexts in the U.S. and elsewhere. Psychologist Diane Ehrensaft defines the gender affirmative model as:

a method of therapeutic care that includes allowing children to speak for themselves about their self‐experienced gender identity and expressions and providing support for them to evolve into their authentic gender selves, no matter at what age. Interventions include social transition from one gender to another and/or evolving gender nonconforming expressions and presentations, as well as later gender‐affirming medical interventions (puberty blockers, cross‐sex hormones, surgeries).

(Ehrensaft 2017, p. 62)

To facilitate this process, the therapist ‘assesses a child’s gender status’ using assessment instruments, observation, play, interviewing, dialogue, or projective measures. Diane Ehrensaft sums up the gender affirmative approach in the following manner: ‘When it comes to knowing a child’s gender, it is not for us to tell, but for the children to say’ (ibid., p. 63).

The affirmative model takes at face value a child or teen’s declaration about feelings and thoughts related to gender. It encourages parents, schools, and other authorities in a child’s life to accept those claims. Though this approach has gained widespread acceptance, I feel that it has significant shortcomings. For example, it may foreclose thinking about a young person’s development by conflating gender dysphoria with trans identification, and it may concretize an adolescent’s desire to transition without extended exploration and assessment. Because the basic premise of the model is that gender is something ‘for the children to say’, unconscious relational, systemic, archetypal and social factors may be denied relevance and exploration (Evans 2020).

Maya came to see me after she had decided to dis‐identify from her trans identification and to re‐identity as female. After getting to know Maya I formed the view that her trans identification was an attempt to adapt to a complex array of interrelated factors: her social environment at school and online; the dynamic between herself and her parents; her rejection of her body inculcated in part by her mother’s insistence on thinness and dieting; and her unmetabolized grief about the loss of a primary attachment figure early in life. In Maya’s case, I understand that affirmative treatment addressed the superficial distress only and seemed to leave little room to explore other factors. It was only after Maya decided to detransition that psychological work in these important areas could occur.

The gender affirmative model of care may leave some or all of these psychological factors unaddressed because one of its basic tenets is that the role of the therapist is to affirm – or confirm – what the patient ‘knows’ (Spiliadis 2019). I believe that this principle is in direct opposition to usual and best psychotherapeutic practice: the role of the therapist is to open space for exploration, for nuanced thinking and expanded and deepened self‐understanding. For Maya, the affirmative approach appears to have supported a narrow and superficial understanding of the presenting problem. According to Maya’s report of this therapeutic encounter, she was encouraged to believe that physical interventions might resolve her conflicting feelings about herself, thereby substituting the possibility of legitimate and potentially transformative ‘suffering’ for a concretized solution that, in my opinion, promoted dissociation from the body.

I have a number of concerns regarding the gender affirmative model of care. It is my view that this model rests on a false premise and encourages the patient to make critical health decisions, including surgical interventions, based on beliefs rather than ‘facts’. Gender identity is not a well‐defined concept and lacks empirical validity. Although there have been efforts to identify biomarkers that might correlate with gender dysphoria, no robust evidence has been found. The U.K.’s Gender Identity Development Service, for example, has noted the chromosomal normality of their patients (Butler et al. 2018).

Gender affirmative clinicians themselves acknowledge the lack of material basis for gender identity. According to advocates of the affirmative approach, ‘we understand gender identity, both its match and its mismatch with assigned natal sex, as primarily informed by a child’s cognitions and emotions, rather than by genitalia and observable external sex characteristics’ (Hidalgo et al. 2013, p. 286). The affirmative approach, then, affirms ‘self‐experienced gender identity’ (Ehrensaft 2017, p. 62) and treats as ‘fact’ a child or young person’s thoughts and feelings about themselves even when those thoughts and feelings do not align with the material reality of the body (Hidalgo et al. 2013, p. 286). I believe that without a basis in biological reality, ‘gender identity’ takes on the significance of an essence, something akin to a soul. The term ‘gender identity’, refers to a person’s inner sense of being a male, female or something else. Whether we believe in the existence of an immortal soul or find it a worthy metaphor for describing experience, we recognize the value of the concept. As a metaphor, the notion of gender identity invites questions about our nature, our relationship with culture, and our relationship with the inner ‘other’. It may be the best possible way to express an ineffable truth, but we do not treat ‘soul’ as an empirical fact. When gender identity is taken as empirical fact, the metaphor becomes concretized and we lose the ability to relate to our inner world in a symbolic way. When metaphors are made literal, the literal body becomes a vehicle for metaphoric expression (Bret Alderman, personal communication).

We can validate a young person’s discomfort with restrictive gender roles. We can celebrate her desire to flout conventional gender norms and to wear clothes or hairstyles that defy gendered expectations. We can affirm and normalize feelings of same‐sex attraction and help her to come to terms with these in a society where homosexuality is not always accepted. We can validate a young person’s distress about the perceived mismatch between her self‐perception and her body. We can affirm the importance of these feelings and the distress that they cause. And we can respect her need to live in the opposite‐sex role as a potential way to manage this distress. However, in my experience, I believe that the gender affirmative model of care perhaps too often confirms prematurely a patient’s belief and forecloses the opportunity for thinking symbolically about this distressing experience. In this case, are we colluding with an avoidance of reality?

## Coming to terms with reality

Jung stressed that adequate adaptation requires that an individual be able to ‘grow in the soil in which it is planted’ before individuation can proceed (1971, para. 761). Adaptation requires confrontation and acceptance of the realities in which we find ourselves. In adolescence, one confronts the end of childhood and begins to meet the demands of the external world. Jung recognized that engaging reality on its own terms was an integral part of this passage:

For most people it is the demands of life which harshly put an end to the dream of childhood. If the individual is sufficiently well prepared, transition to a profession or career can take place smoothly. But if he clings to illusions that are contrary to reality, then problems will surely arise.

(Jung 1960, para. 761)

Livia, a 23‐year‐old detransitioned woman who had a mastectomy and hysterectomy when she was 20 and 21 respectively spoke at the Detransition Advocacy Network event in Manchester, U.K. in 2019. She emphasized the role of reality in her transition and detransition:

It’s really hard to focus on one thing but the word that’s stuck in my mind the most is ‘reality.’ I feel like for me transition was a way to get out of my reality as a homosexual woman ….

When we started this conversation the word that was important to me was ‘reality’. And reality to me is that… a hysterectomy and removal of your ovaries doesn’t make you any less female. So, it doesn’t make any sense to me why this is called transition or a sex change because it’s not it’s castration. And now that I am trying to care for my health as much as possible, I spend a lot of time on hysterectomy support sites and message boards for women – for women because only women get hysterectomies, and only women deal with the consequences of a hysterectomy.

(Livia 2019)

Affirmative care for teens may inadvertently reify ‘defenses against reality’ (Lemma 2016, p. 366). Life’s task – as well as the task of analysis – is to come to terms with that which cannot be changed and to mourn this so that we may move forward. The reality of our bodies insists on being confronted and accepted in adolescence. This task can be particularly challenging for adolescent females. Social and medical transition undertaken without adequate understanding may facilitate a ‘bypassing of a mourning process about that which cannot be changed’ (ibid., p. 369).

Coming to terms with bodily reality is a major task in adolescence. It is the time when we learn what our adult body will look like. How tall will I be? How will I look? Will I tend to put on weight easily? The inexorable reality of our bodies is, for many of us, the first demand to face limitations, a painful prerequisite for coming into existence in three‐dimensional space and time. Jung understood the great importance of coming to terms with reality – and the reality of our bodies – as regards individuation:

If you were a spirit you could be anywhere, but the damnable fact is that you are rooted just here, and you cannot jump out of your skin; you have definite necessities. You cannot get away from the fact of your sex, for instance, or of the colour of your eyes, or the health or the sickness of your body, your physical endurance. Those are definite facts which make you an individual, a self that is just yourself and nobody else. If you were a spirit you could exchange your form every minute for another one, but being in the body you are caught; therefore, the body is such an awkward thing: it is a definite nuisance. All people who claim to be spiritual try to get away from the fact of the body; they want to destroy it in order to be something imaginary, but they never will be that, because the body denies them; the body says otherwise. They think they can live without sex or feeding, without the ordinary human conditions; and it is a mistake, a lie, and the body denies their convictions.

(Jung 1988, pp. 63‐64).

Agonists that suppress the release of natural hormones, cross‐sex hormones that create opposite‐sex characteristics, and surgery that removes body parts or creates facsimiles of them can be seen as Promethean efforts to subdue biological reality. We defy the body at a cost. As Jung says, we must acknowledge the realities of embodiment.

## Reality and trauma

Psychological trauma warps our sense of reality, rendering us unable to trust our senses. Helping an analysand to reclaim her relationship with reality can be an important aspect of trauma work. Maya’s grief over the loss of her aunt was denied and she was encouraged to ‘move on’, as though that relationship were insignificant. The reality of her relationship with her parents had also not been mirrored back to her. This rupture in her sense of reality made it difficult for her to make meaning out of her situation and contributed to poor affect regulation as she was always confused about the nature and location of the problem. When Maya detransitioned and reaffirmed her female identity and accepted her biological reality, her distress decreased, and she felt somewhat more contained. As she reconnected with reality in her relationships, her sense of coherence and psychic equilibrium increased. Even though reality was distressing, having a firm grasp on her emotional truth gave her greater resilience.

The case of Maya illustrates the extent to which her presenting problem was a metaphor for unresolved grief and deficient parenting which was later exacerbated by both peer and, in my opinion, professional wounding. The purpose of psychological treatment is to bring unconscious issues to consciousness, thereby recovering and reconnecting affect, cognition and reality. Depth psychology posits the active presence of unconscious compensation and symbolization processes. We owe it to young people to explore multiple facets of any individual’s expressed desire to transition. For some people, living life in the opposite sex role – even to the point of undergoing physical transition – may be what the psyche requires of them. We help our patients best by affirming the significance of their experience but without explicitly endorsing a specific course of action.

## The mythos of gender identity

If, as I do, we think of a belief in gender identity as a kind of neurotic fantasy, ‘we might also imagine a hidden treasure within it, something potentially curative and redemptive’ (Alderman 2016, p. 58). What is this symptom trying to cure or compensate in the collective? My view is that the affirmative model of care concretizes psychic pain, locates it in the body, and seeks biomedical treatments for it. Paradoxically, it also offers a compensatory belief in a disembodied, ineffable essence. In a BBC documentary, transgender psychotherapist Herschel Russell relates the story of a mum who asked her eight‐year‐old gender diverse child how he knew he was really a boy. According to Russell, the child responded, ‘I know way down deep where the music plays’ (Conroy 2017). With the continued decline of traditional religious beliefs, are we unconsciously seeking a new way to conceptualize spirit, and does gender as a mysterious, disembodied imperative offer this? The mythos that informs affirmative care paradoxically allows us to see ourselves as beings with an existence that transcends our mere corporeal form. Jung famously remarked that ‘the gods have become diseases’ (1967, para. 54). I have wondered if gender identity theory may be an unconscious attempt to find the divine hidden within the disease. We are perhaps being asked to find a new a connection with that which is nonrational and transcends materiality. If this is the corrective offered by gender identity theory, we would do well to allow ourselves to be informed by this impulse while also maintaining contact with embodied reality.
